# The Behaviour of Home Advantage during the COVID-19 Pandemic in European Rink Hockey Leagues

**DOI:** 10.3390/ijerph19010228

**Published:** 2021-12-26

**Authors:** Jordi Arboix-Alió, Guillem Trabal, Bernat Buscà, Javier Peña, Adrià Arboix, Raúl Hileno

**Affiliations:** 1 Department of Sports Science, Ramon Llull University, FPCEE Blanquerna, 08025 Barcelona, Spain; bernatbs@blanquerna.url.edu; 2 Department of Physical Activity Sciences, University of Vic—Central University of Catalonia, 08500 Vic, Spain; guillem.trabal@uvic.cat (G.T.); javier.pena@uvic.cat (J.P.); 3 Sport Performance Analysis Research Group (SPARG), University of Vic—Central University of Catalonia, 08500 Vic, Spain; 4 Sport and Physical Activity Studies Centre (CEEAF), University of Vic—Central University of Catalonia, 08500 Vic, Spain; 5 Cerebrovascular Division, Department of Neurology, Hospital Universitari del Sagrat Cor, Universitat de Barcelona, 08029 Barcelona, Spain; aarboix@quironsalud.es; 6 National Institute of Physical Education of Catalonia (INEFC), University of Lleida, 25192 Lleida, Spain; rhileno@gencat.cat

**Keywords:** roller hockey, coronavirus, match variables, performance analysis, crowd behaviour

## Abstract

The primary purpose of the present study was to compare the home advantage (HA) and the home team performance in the most relevant European rink hockey leagues (Spanish, Portuguese and Italian), considering the presence or absence of spectators in the competition venues due to the effect of COVID-19 restrictions. The sample was composed of 1665 rink hockey matches (654 from the Spanish league, 497 from the Portuguese league, and 514 from the Italian league) played between the 2018–2019 and 2020–2021 seasons. The HA and match variables comparisons were established using several negative binomial regression models. Results showed that the effect of HA did not disappear despite playing without spectators but decreased from 63.99% to 57.41% (*p* = 0.002). Moreover, the comparison of the match variables showed that playing with spectators benefited local teams’ performance, especially in the Portuguese and Italian leagues. Playing with spectators favoured local team performance in rink hockey matches, which is more evident in some analysed leagues. However, as HA does not disappear entirely without spectators, it is necessary to study other relevant performance factors that are not directly or indirectly attributable to crowd behaviour in rink hockey performance analyses.

## 1. Introduction

In recent years, the increasing interest in sports performance analysis has resulted in many studies regarding match variables in team sports [1]. Rink hockey, also known as roller hockey or hardball hockey, is not an exception, and, lately, the number of studies about this sport has grown considerably [2].

One of the most studied match variables in sports science is the effect of match location [3]. This concept is known as the ’home advantage’ (HA) effect and may be defined as home teams’ advantage over the visiting team by playing in their home court [4]. This phenomenon was firstly studied by Schwartz and Barsky [5] in different team sports, such as basketball, ice hockey, American football, or baseball in the United States.

HA has been widely studied and documented in a variety of different sports, competition standards and countries [6] in individual disciplines such as tennis [7], judo [8,9], speed skating [10], boxing [11] or golf [12], and team sports such as football [13,14], basketball [15,16], rugby [17,18], handball [19,20] and water polo [3]. HA can be affected by the format of the competition, showing differences depending on whether the competition is a playoff, a knockout or a regular league [21,22,23]. Although HA influences differently depending on the sport, region, or competitive standard, it can be quantified by 60% on average [24]. To our knowledge, only two studies have previously focused on HA in rink hockey, determining a 59.80% and 60.88% advantage in Spanish and Portuguese leagues, respectively [25,26].

The adverse effects of travel fatigue, the familiarity with the context, the referee bias, the territoriality, the rules that favour the home team, or the effect of the home crowd have been identified as explaining factors for the HA phenomenon [27]. According to Pollard et al. [28], the majority of the HA studies have tended to consider each factor in isolation when attempting to explain the HA effect. However, determining how these factors operate and how they affect performance is still unclear. In this vein, there is still little consensus about the weight of each factor in the HA effect, which is currently considered a multifactorial phenomenon with a variety of interacting causes and contributing factors [29]. Among them, the crowd effect is one of the most studied. It is suggested that crowd support could influence team performance by placing home players in a more positive and confident psychological state [30]. Thus, Schwartz and Barsky [5] found that crowd density increased the HA in Major League baseball. HA increased from 48% in relatively empty venues (less than 20% capacity) to 55% when the venues were between 20 and 40% of their capacity, and the value was 57% when crowd density was greater than 40% capacity.

Similarly, Agnew and Carron [31] showed crowd density to be significantly related to the HA in ice hockey matches (R^2^ = 0.011, *p* < 0.001). Furthermore, the public could influence not only the players but also the referees, creating a bias in favour of the home team [32], receiving significantly fewer penalties and disciplinary cards than the visiting team [32,33]. In this vein, Nevill et al. [30] noted that a large audience and noise generation could lead to an imbalance in referees’ decisions in favour of the home team.

Currently, given the unprecedented times we face because of the global COVID-19 pandemic, many sporting events have been played without an audience, a move that has affected team-sport leagues, including rink hockey. This situation creates a unique and natural scenario to study crowd influence and compare the HA phenomenon with spectators and without them [34].

To the best of our knowledge, no previous research has analysed the influence of crowd support in rink hockey in the above mentioned terms. Thus, the primary purpose of this study was to analyse the effect of the absence or presence of spectators in HA and other relevant match variables. It was hypothesized that the HA effect will be lower (fewer points) without home supporters at the competition venue and that visiting teams will benefit from this circumstance.

## 2. Materials and Methods

### 2.1. Sample

In order to carry out the study, 1665 rink hockey matches were analysed: *OkLiga* (Spanish league; 654 matches), *1a Divisao* (Portuguese league; 497 matches), and *Serie A1* (Italian league; 514 matches). These rink hockey leagues have a similar competition format; each team plays every other team once at home and once away during the season. Only regular-season matches have been included in the sample. In all played matches, there was a home and a visiting team. The scoring system of all the analysed rink hockey leagues was: 3 points for a win, 1 point for a draw, and 0 points for a loss. This league structure allows an unbiased method for quantifying the HA over a complete season [35].

### 2.2. Design and Procedures

The dataset of this study was collected through the open-access websites from each Rink Hockey Federation. Additionally, match data were rechecked and validated by using the independent website hockeypista.it (http://www.hockeypista.it, accessed on 26 November 2021). Before data collection, written permission from all the website administrators was received, with the respective privacy policies being entirely respected. The methodological procedures conformed to the ethics guidelines of a local university, and the investigation was conducted in compliance with the principles expressed in the Declaration of Helsinki (revised in Fortaleza) [36].

### 2.3. Variables

Table 1 shows the different analysed variables.

### 2.4. Statistical Analysis

The causal effect of the presence of spectators on the number of goals scored, the number of set-pieces shot, the number of faults committed, and the number of cards received was quantified using several negative binomial regression models—suitable models for the analysis of count-dependent variables exhibiting the phenomenon of overdispersion [37]. A dependent variable Y (goals scored, set-pieces shot, faults committed, or cards received), an independent variable X (spectators), and two moderator variables M (match location and opponent’s level) were included in each model. Moderator variables were also included as adjustment variables by the hierarchical principle. The exposure time *t* was not included in the models because all the matches analysed lasted the same time (50 min). Therefore, the multiplicative formulation of the negative binomial models constructed was as follows:

$${\tilde{\mu }}_{i}=e^{\left(\beta_{0}+\beta_{1}\times Spect+\beta_{2}\times MatLoc+\beta_{3}\times OppLev+\beta_{4}\times Spect\times MatLoc+\beta_{5}\times Spect\times OppLev+\varepsilon_{i}\right)}$$

The factor change $e^{\beta_{1}}$ was expressed as a percentage change in the expected mean count of Y for one-unit increase in X with the following formula:

$$\left[e^{|\beta_{1}|\times 1}-1\right]\times 100$$

The goodness-of-fit of the binomial regression models constructed was assessed by analysing the deviance residuals [38]. These residuals were represented by box plots and checked whether they were all within the interval −2 to 2.

The presence of overdispersion was tested using different procedures exposed by Long [39], Doménech and Navarro [40]. There was the problem of overdispersion (a) if the quotient between variance and mean of each count-dependent variable was greater than 1, (b) if the quotient between deviance and residual degrees of freedom (−2LL/df_Res_) of each Poisson model was statistically greater than 1 (procedure only applicable when all predictors are categorical), or (c) if the likelihood-ratio test of the parameter α = 0 was statistically significant.

Statistical analyses were performed using Stata/IC v.17.0 statistical package (Stata Corporation, College Station, TX, USA). Analyses were performed on all sample data and stratified by the European national league (Spanish *OkLiga*, Portuguese *CN 1ª Divisão*, and Italian *Serie A1*). The significance level was set at *p* < 0.05 for all tests.

## 3. Results

**Figure 1:**
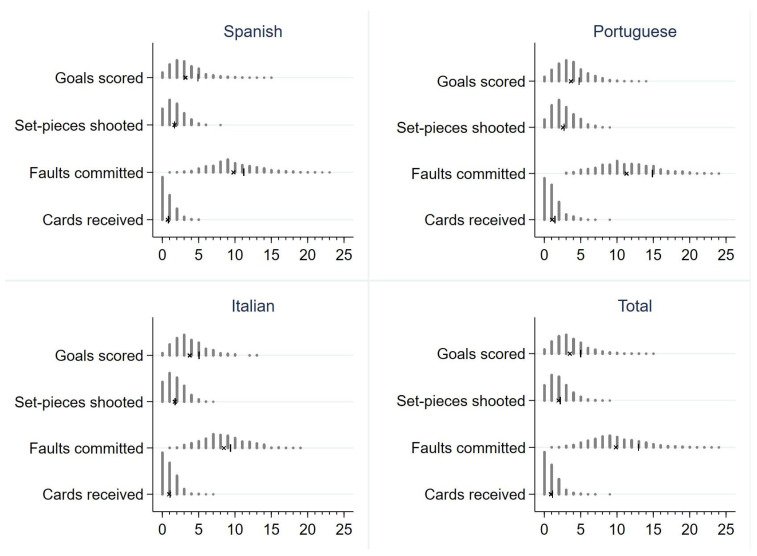
Distribution of the count-dependent variables.

Figure 1 shows the distribution of the count-dependent variables. The goals scored, set-pieces shot, and cards received variables presented a negative binomial distribution: a right-skewed distribution with a predominance of zero and near-zero values; and a variance greater than its mean (symbol | and X in the figure, respectively). Conversely, the faults committed variable presented an approximation of the negative binomial distribution to the normal distribution because its mean was higher than the other variables.

**Figure 2:**
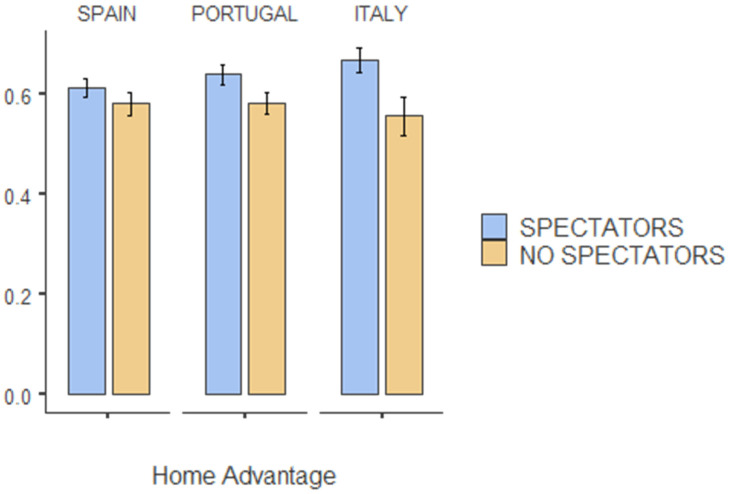
Home Advantage values in the Spanish, Portuguese and Italian leagues in games with and without spectators.

The descriptive analysis of match variables and the comparison of mean pre- and post-COVID are presented in Table 2. In local teams, the only significant difference when playing without public was a higher number of cards received (0.91 ± 1.03 vs. 0.76 ± 0.93; *p* = 0.003). However, local teams were sanctioned with more defensive fouls and scored fewer goals when playing without spectators despite not being significant. Conversely, the visiting teams significantly scored more goals when playing with no public (3.40 ± 2.10 vs. 3.03 ± 1.91; *p* > 0.001). As for the HA, Figure 2 clearly shows how the sample value of “points HA%” = 57.41 falls far below the value of 63.99 (*p* = 0.002). This decrease in the HA value is especially evident in the Italian League (66.74% vs. 55.73%; *p* = 0.016). In the Portuguese and Spanish leagues, despite not being significant, there also exists an HA decrease (64.01% vs. 58.31%; *p* = 0.09 and 61.41% vs. 58.10%; *p* = 0.284, respectively).

Table 3 shows the number of goals scored with and without spectators, according to match location and opponent’s level. The results showed that visiting teams, playing with spectators (relative to playing without) decreased the expected mean number of goals scored by a significant factor of 0.90 (95% CI: 0.84, 0.96; *p* = 0.002), both when the match was played against a lower-level opponent and when it was played against a higher-level opponent (*p* = 0.01; 95% CI: 0.83, 0.97).

Table 4 shows the number of set-pieces shot with and without spectators, according to match location and opponent’s level. Results show that visiting teams, when playing with spectators (relative to playing without), decreased the expected mean number of set-pieces shot by a significant factor of 0.87 (95% CI: 0.79, 0.95; *p* = 0.003) and 0.85 (95% CI: 0.77, 0.93; *p* = 0.001) both versus higher and lower opponents, respectively.

Table 5 shows the number of defensive faults with and without spectators, according to match location and opponent’s level. Results showed that playing against a lower level team with audience (relative to playing without), decreased the expected mean number of defensive faults by a significant factor of 0.95 (95% CI: 0.91, 1.00; *p* = 0.03) and 0.95 (95% CI: 0.91, 1.00; *p* = 0.037).

Table 6 shows the number of cards received with and without spectators, according to match location and opponent’s level. Results show that when teams play at home without spectators (relative to playing with) increase the number of received cards by a significant factor of 0.82 (95% CI: 0.70, 0.96; *p* = 0.012) and 0.85 (95% CI: 0.73, 0.98; *p* = 0.025).

## 4. Discussion

The primary purpose of the present study was to analyse the differences in the HA effect and several match variables in European rink hockey leagues in matches with and without spectators. The main finding was that the HA value decreased when local teams played without spectators, and some match variables modified their behaviour. This piece is the first study to analyse the influence of crowd presence or absence in rink hockey to the best of our knowledge. However, despite the lack of available studies to compare the present results, these findings align with previous team-sport studies reporting a decrease in the HA values or several match variables when playing without spectators [29,38,39].

### 4.1. Home Advantage and Scoring

Data revealed that the HA effect remains in rink hockey despite playing without spectators since the local teams still achieved a higher percentage of the disputed points (57.41%). However, this advantage was lower than the observed in the same leagues when games were played with spectators sitting on the stands (63.99%). Similarly, Van de Ven [41] examined twenty football matches played behind closed doors and found that the HA remains present. In the same vein, Ponzo and Scoppa [32] examined a higher number of derbies whose teams played in the same stadium and found a significant HA effect, thus attributing this result mainly to the noise coming from the crowd. Moreover, Scopa [42] reported that psychological elements and the social context could strongly affect individual performance and decision-making. Thus, the crowd may directly influence players by encouraging their team or intimidating the opponent and indirectly influencing the referees’ decisions. Neville et al. [30] analysed the effect of crowd noise upon refereeing decisions in 40 English football referees. They had to referee a pre-recorded match between Liverpool (local) and Leicester (visitant). The referees were randomly distributed into two groups: (a) group with ambient noise from the field and (b) group without noise. Group (a) reported 15.5% fewer home team defensive fouls than group (b). Furthermore, the responses of the group (a) were almost identical to those of the match referee.

Regarding the goals scored in the present study, as a general trend, visitant teams performed better when played without spectators and scored more goals. Conversely, local teams had a worse performance when played without public and scored fewer goals despite not being significant. This trend is especially evident in the Portuguese league, where local teams scored 11.6% more goals and in the Italian league, where visitant teams scored −24.3% goals when playing with spectators. Surprisingly, no significant differences were found in the number of goals between playing with and without spectators in the Spanish league. These differences could be explained because of the higher spectator attendance in the Italian and Portuguese championships. It has been estimated that the goal difference between locals and visitors increases by 0.1 goals per every 10,000 spectators in football [43].

### 4.2. Disciplinary Cards and Defensive Faults

Local teams received 21.8% fewer disciplinary cards when playing in their supporters’ presence than without spectators. In this regard, there were also differences between the Portuguese and the Spanish leagues, which could be explained again by the average numbers of competition attendance.

The results showed that teams committed fewer faults when playing with spectators, both at home (5.2%) and away (5%). Surprisingly, match location does not influence the number of defensive faults. However, these differences were only significant when teams played against an opponent with a lower level, probably because the superior team uses a riskier and more aggressive style in these matches, thus pressing its opponents throughout the entire court and consequently having more chances to commit fouls. This particular case scenario is very common in rink hockey, where there is evident bias caused by the different budgets of teams competing in the same division [44]. This issue causes more level heterogeneity than in other team sports with professional and semi-professional athletes in the same competitions [45]. For this reason, among the different situational variables, the level of teams has been reported as one of the most decisive variables in rink hockey match outcomes [46].

### 4.3. Individual Set-Pieces

Individual set-pieces are probably one of the most relevant aspects influencing the match outcome in rink hockey [47]. These set-pieces are particular events involving a direct opposition between the shooter and the goalkeeper and are conformed for free direct hits and penalties. In free direct hits, the shooter has five seconds to start the execution (from 7.4 m), being able to choose a direct shot or approaching and dribbling towards the goalkeeper to score, while in the PEN, the shooter has five seconds to start the execution, consisting of a direct shot on goal from the penalty point (5.4 m) [48]. Both set-pieces happen when a player commits a defensive fault in a manifest goal action (the penalty happens inside the area). Moreover, a free direct hit happens when a player is sanctioned with a blue card or when a team accumulates 10 defensive faults.

The present results demonstrated that visitant teams shot a higher significant number of set-pieces per match when playing without spectators compared to playing with the local crowd. However, local teams also shot more set-pieces without spectators. It is challenging to elucidate the reason for these results. It can be hypothesized that the referees feel less pressure by not having the crowd in the stands and, therefore, feel less pressure to call a penalty or a free direct hit. Likewise, it cannot be ruled out that inactivity during the lockdowns has altered the players’ physical fitness [49]. This particular circumstance could result in players tackling less precisely, causing a higher number of punishable actions.

Despite the usefulness of these findings, the present study has some limitations which must be acknowledged and addressed in future studies. Firstly, the lack of studies about rink hockey to establish comparisons reduces the possibility to identify some tendencies between findings. Secondly, we did not consider factors that might contribute to HA, such as travel fatigue for the visitants or within-game events. Moreover, factors such as the playing surface (wood, terrazzo, or synthetic), court dimensions, or differences in temperature and humidity (which could alter the grip of the soil) may also be relevant to explain the HA. Another essential aspect that was not possible to consider is referees’ anxiety, which probably plays a role in their decisions given these findings. Finally, it would be pertinent to study in-depth other aspects related to the crowd behaviour that have not been considered in this piece of research, such as the involvement in encouraging their team, the aggressiveness towards the opponents, or crowd density. The strength of the present study lies in the number of analyzed matches without spectators of the most prestigious rink hockey leagues and, for its novelty, being the first study to analyse the crowd influence in this sport..

## 5. Conclusions

In conclusion, the current study provided more evidence for the complex HA puzzle, reporting new data regarding the influence of spectators in a different discipline. Playing with spectators benefits local teams’ performance, which is more evident in the Portuguese and the Italian leagues. However, as the HA does not disappear without home crowds, other contributing factors are not directly or indirectly attributable to a significant number of spectators on the stands. Therefore, there are probably multiple and complementary reasons that would also explain the better performance on the home court and playing without spectators such as court dimensions, playing surface or travel fatigue.

The present results could help prepare players with specific psychological exercises focusing on the game and not being influenced by the crowd’s presence. These could include attempts to increase familiarity in home matches, reduce travel exhaustion, stimulate territoriality or ensure that player expectation and tactical adjustments in away matches do not hinder success. Likewise, these results can also be relevant to referees aiming for objective decision making or team officials, coaches, and players trying to influence home advantage to their advantage. In this vein, the referees, who are expected to be objective in their decisions, should be specifically trained to fulfil their objective even under pressure. We hope that our research can help to encourage similar studies that can clarify the relationship between HA and the crowd effect.

## Figures and Tables

**Table 1:** Properties of the analysed variables.

Role	Variable	Category (Code)	Description
Independent variable	spectators	No (0)	The match was played without spectators.
		Yes (1)	There match was played with spectators.
Dependent variable	goals scored		Number of goals scored per match.
	Individual set-pieces shot		Number of individual set-pieces shot per match.
	faults committed		Number of disciplinary faults committed by players per match played.
	cards received		Number of disciplinary cards (blue and red) received by players per match played.
Moderator variable	match location	Away (0)	The analysed team played away.
		Home (1)	The analysed team played at home.
	opponent’s level	Lower (0)	The opponent finished the league in a lower position.
		Higher (1)	The opponent finished the league in a higher position.

**Table 2:** Descriptive analysis of match variables according to match location and spectators presence. The data represents events per match for each team and are shown as mean ± SD.

	Home			Away		
	Spectators	No Spectators	*p*	Spectators	No Spectators	*p*
Goals	3.89 ± 2.48	3.77 ± 2.24	0.338	3.03 ± 1.91	3.40 ± 2.10	>0.001
Individual set-pieces	0.72 ± 0.90	0.80 ± 0.86	0.104	0.54 ± 0.73	0.59 ± 0.72	0.221
Faults committed	9.73 ± 3.52	10.02 ± 3.56	0.121	9.74 ± 3.66	10.05 ± 3.66	0.109
Cards	0.76 ± 0.93	0.91 ± 1.03	0.003	0.98 ± 1.14	0.97 ± 1.13	0.854

**Table 3:** Effect of playing with spectators on the number of goals scored in different leagues and competitive situations.

League	Match Location	Opponent’s Level	Spectators	$\tilde{\mu }$	$e^{\beta }$	*%*	*p*
Spanish	Away	Lower	No	3.94 [3.54, 4.38]	0.92 [0.81, 1.04]	−9.1 [−23.5, 3.8]	0.169
(*n* = 1308)			Yes	3.61 [3.38, 3.85]			
		Higher	No	2.17 [1.91, 2.46]	0.89 [0.77, 1.03]	−12.3 [−30.3, 3.3]	0.125
			Yes	1.93 [1.79, 2.08]			
	Home	Lower	No	4.66 [4.20, 5.17]	0.97 [0.86, 1.09]	−2.8 [−15.8, 9.5]	0.643
			Yes	4.53 [4.27, 4.81]			
		Higher	No	2.57 [2.28, 2.90]	0.94 [0.82, 1.09]	−5.9 [−21.9, 8.7]	0.424
			Yes	2.42 [2.26, 2.61]			
Portuguese	Away	Lower	No	4.09 [3.73, 4.48]	1.03 [0.92, 1.15]	3.0 [−8.8, 15.5]	0.607
(*n* = 994)			Yes	4.21 [3.93, 4.51]			
		Higher	No	2.43 [2.18, 2.71]	1.01 [0.88, 1.15]	0.9 [−13.3, 15.4]	0.893
			Yes	2.45 [2.26, 2.66]			
	Home	Lower	No	4.71 [4.32, 5.13]	1.12 [1.00, 1.24]	11.6 [0.4, 24.2]	0.043
			Yes	5.25 [4.94, 5.59]			
		Higher	No	2.80 [2.52, 3.11]	1.09 [0.96, 1.24]	9.3 [−4.0, 24.3]	0.174
			Yes	3.06 [2.84, 3.30]			
Italian	Away	Lower	No	4.85 [4.45, 5.29]	0.80 [0.72, 0.90]	−24.3 [−39.0, −11.1]	<0.001
(*n* = 1028)			Yes	3.90 [3.64, 4.19]			
		Higher	No	2.81 [2.54, 3.12]	0.88 [0.77, 1.00]	−14.1 [−30.0, −0.1]	0.048
			Yes	2.47 [2.28, 2.67]			
	Home	Lower	No	5.00 [4.59, 5.45]	1.02 [0.92, 1.14]	2.5 [−8.5, 14.0]	0.651
			Yes	5.13 [4.82, 5.46]			
		Higher	No	2.90 [2.62, 3.21]	1.12 [0.98, 1.27]	11.7 [−1.7, 26.8]	0.089
			Yes	3.24 [3.00, 3.49]			
Total	Away	Lower	No	4.30 [4.07, 4.55]	0.90 [0.84, 0.96]	−11.4 [−19.3, −4.1]	0.002
(*n* = 3330)			Yes	3.86 [3.71, 4.02]			
		Higher	No	2.48 [2.33, 2.65]	0.90 [0.83, 0.97]	−11.1 [−20.4, −2.6]	0.010
			Yes	2.23 [2.13, 2.34]			
	Home	Lower	No	4.79 [4.54, 5.06]	1.02 [0.96, 1.09]	2.3 [−4.2, 9.1]	0.484
			Yes	4.90 [4.73, 5.08]			
		Higher	No	2.76 [2.59, 2.94]	1.03 [0.95, 1.11]	2.6 [−5.2, 10.8]	0.509
			Yes	2.84 [2.72, 2.96]			

Abbreviation: 95% confidence intervals in brackets. *n* = number of observations; $\tilde{\mu }$ = expected mean number of goals scored; $e^{\beta }$ = factor change in the expected mean number of goals scored when moving from playing without spectators to playing with spectators, holding other variables constant; % = percentage change in the expected mean number of goals scored when moving from playing without spectators to playing with spectators, holding other variables constant; *p* = *p*-value for *z*-test.

**Table 4:** Effect of playing with spectators on the number of set-pieces shot in different leagues and competitive situations.

League	Match Location	Opponent’s Level	Spectators	$\tilde{\mu }$	$e^{\beta }$	*%*	*p*
Spanish	Away	Lower	No	2.10 [1.85, 2.39]	0.74 [0.63, 0.86]	−35.9 [−58.9, −16.2]	<0.001
(*n* = 1308)			Yes	1.55 [1.42, 1.69]			
		Higher	No	1.78 [1.54, 2.04]	0.71 [0.60, 0.83]	−41.8 [−67.7, −19.9]	<0.001
			Yes	1.25 [1.14, 1.37]			
	Home	Lower	No	2.29 [2.02, 2.61]	0.81 [0.70, 0.95]	−23.1 [−43.3, −5.8]	0.007
			Yes	1.86 [1.72, 2.02]			
		Higher	No	1.94 [1.70, 2.21]	0.78 [0.66, 0.91]	−28.5 [−50.8, −9.6]	0.002
			Yes	1.51 [1.38, 1.65]			
Portuguese	Away	Lower	No	2.29 [2.04, 2.58]	1.02 [0.88, 1.18]	2.0 [−13.5, 18.2]	0.788
(*n* = 994)			Yes	2.34 [2.14, 2.56]			
		Higher	No	2.11 [1.87, 2.38]	1.07 [0.92, 1.24]	6.9 [−8.6, 24.0]	0.384
			Yes	2.26 [2.07, 2.47]			
	Home	Lower	No	2.73 [2.44, 3.05]	1.11 [0.97, 1.27]	10.7 [−3.5, 26.9]	0.144
			Yes	3.02 [2.79, 3.27]			
		Higher	No	2.51 [2.24, 2.82]	1.16 [1.01, 1.33]	15.9 [0.8, 33.4]	0.039
			Yes	2.91 [2.69, 3.16]			
Italian	Away	Lower	No	1.74 [1.52, 1.99]	0.90 [0.75, 1.06]	−11.6 [−32.6, 6.4]	0.209
(*n* = 1028)			Yes	1.56 [1.40, 1.73]			
		Higher	No	1.67 [1.45, 1.91]	0.84 [0.70, 1.00]	−19.2 [−41.9, −0.1]	0.048
			Yes	1.40 [1.26, 1.56]			
	Home	Lower	No	2.03 [1.79, 2.31]	0.92 [0.78, 1.08]	−8.4 [−27.4, 8.4]	0.325
			Yes	1.87 [1.70, 2.07]			
		Higher	No	1.95 [1.71, 2.22]	0.86 [0.73, 1.02]	−15.8 [−36.7, 2.0]	0.084
			Yes	1.68 [1.52, 1.87]			
Total	Away	Lower	No	2.04 [1.89, 2.21]	0.87 [0.79, 0.95]	−15.5 [−26.9, −5.1]	0.003
(*n* = 3330)			Yes	1.77 [1.67, 1.87]			
		Higher	No	1.86 [1.72, 2.01]	0.85 [0.77, 0.93]	−17.9 [−29.9, −7.0]	0.001
			Yes	1.57 [1.49, 1.67]			
	Home	Lower	No	2.35 [2.19, 2.53]	0.93 [0.85, 1.02]	−7.6 [−17.7, 1.6]	0.108
			Yes	2.19 [2.08, 2.30]			
		Higher	No	2.14 [1.98, 2.30]	0.91 [0.83, 1.00]	−9.8 [−20.5, −0.1]	0.047
			Yes	1.95 [1.84, 2.05]			

Abbreviation: 95% confidence intervals in brackets. *n* = number of observations; $\tilde{\mu }$ = expected mean number of set-pieces shot; $e^{\beta }$ = factor change in the expected mean number of set-pieces shot when moving from playing without spectators to playing with spectators, holding other variables constant; % = percentage change in the expected mean number of set-pieces shot when moving from playing without spectators to playing with spectators, holding other variables constant; *p* = *p*-value for *z*-test.

**Table 5:** Effect of playing with spectators on the number of faults committed in different leagues and competitive situations.

League	Match Location	Opponent’s Level	Spectators	$\tilde{\mu }$	$e^{\beta }$	*%*	*p*
Spanish	Away	Lower	No	10.06 [9.45, 10.71]	0.96 [0.89, 1.03]	−4.6 [−12.5, 2.8]	0.229
(*n* = 1308)			Yes	9.62 [9.27, 9.99]			
		Higher	No	9.97 [9.35, 10.63]	0.99 [0.92, 1.06]	−1.4 [−9.1, 6.2]	0.721
			Yes	9.84 [9.48, 10.21]			
	Home	Lower	No	10.33 [9.69,11.00]	0.92 [0.85, 0.99]	−9.0 [−17.3, −1.2]	0.022
			Yes	9.48 [9.13, 9.84]			
		Higher	No	10.24 [9.62, 10.90]	0.95 [0.88, 1.02]	−5.6 [−13.5, 1.8]	0.139
			Yes	9.69 [9.34, 10.06]			
Portuguese	Away	Lower	No	11.47 [10.80, 12.19]	1.01 [0.93, 1.08]	0.5 [−7.3, 8.5]	0.894
(*n* = 994)			Yes	11.53 [11.01, 12.08]			
		Higher	No	11.04 [10.39, 11.74]	1.02 [0.94, 1.10]	2.0 [−5.9, 10.1]	0.619
			Yes	11.26 [10.75, 11.79]			
	Home	Lower	No	11.51 [10.83, 12.23]	0.99 [0.92, 1.07]	−1.2 [−9.2, 6.6]	0.752
			Yes	11.37 [10.86, 11.90]			
		Higher	No	11.08 [10.42, 11.77]	1.00 [0.93, 1.08]	0.2 [−7.8, 8.2]	0.959
			Yes	11.10 [10.59, 11.63]			
Italian	Away	Lower	No	9.13 [8.58, 9.72]	0.89 [0.82, 0.96]	−12.2 [−21.4, −3.7]	0.004
(*n* = 1028)			Yes	8.14 [7.75, 8.54]			
		Higher	No	8.60 [8.07, 9.17]	0.96 [0.88, 1.03]	−4.6 [−13.3, 3.5]	0.266
			Yes	8.23 [7.84, 8.63]			
	Home	Lower	No	8.76 [8.22, 9.33]	0.97 [0.89, 1.05]	−3.5 [−12.0, 4.6]	0.398
			Yes	8.47 [8.08, 8.87]			
		Higher	No	8.25 [7.74, 8.80]	1.04 [0.96, 1.12]	3.7 [−4.5, 12.3]	0.379
			Yes	8.56 [8.15, 8.98]			
Total	Away	Lower	No	10.22 [9.85, 10.61]	0.95 [0.91, 1.00]	−5.2 [−10.1, −0.5]	0.030
(*n* = 3330)			Yes	9.72 [9.47, 9.98]			
		Higher	No	9.87 [9.50, 10.26]	0.99 [0.94, 1.04]	−1.1 [−5.9, 3.6]	0.633
			Yes	9.76 [9.51, 10.02]			
	Home	Lower	No	10.19 [9.82, 10.59]	0.95 [0.91, 1.00]	−5.0 [−10.0, −0.3]	0.037
			Yes	9.71 [9.46, 9.96]			
		Higher	No	9.84 [9.48, 10.22]	0.99 [0.95, 1.04]	−1.0 [−5.7, 3.7]	0.683
			Yes	9.75 [9.50, 10.01]			

Abbreviation: 95% confidence intervals in brackets. n = number of observations; $\tilde{\mu }$ = expected mean number of faults committed; $e^{\beta }$ = factor change in the expected mean number of faults committed when moving from playing without spectators to playing with spectators, holding other variables constant; % = percentage change in the expected mean number of faults committed when moving from playing without spectators to playing with spectators, holding other variables constant; *p* = *p*-value for *z*-test.

**Table 6:** Effect of playing with spectators on the number of cards received in different leagues and competitive situations.

League	Match Location	Opponent’s Level	Spectators	$\tilde{\mu }$	$e^{\beta }$	*%*	*p*
Spanish	Away	Lower	No	0.88 [0.71, 1.09]	0.68 [0.53, 0.89]	−46.3 [−89.8, −12.9]	0.004
(*n* = 1308)			Yes	0.60 [0.52, 0.70]			
		Higher	No	1.00 [0.81, 1.23]	0.84 [0.65, 1.07]	−19.4 [−53.2, 7.5]	0.164
			Yes	0.84 [0.73, 0.95]			
	Home	Lower	No	0.82 [0.66, 1.03]	0.64 [0.49, 0.84]	−56.5 [−104.9, −19.5]	0.001
			Yes	0.53 [0.45, 0.61]			
		Higher	No	0.93 [0.75, 1.15]	0.78 [0.61, 1.01]	−27.7 [−64.4, 0.9]	0.058
			Yes	0.73 [0.63, 0.84]			
Portuguese	Away	Lower	No	0.89 [0.72, 1.09]	1.43 [1.11, 1.84]	42.7 [10.7, 84.0]	0.006
(*n* = 994)			Yes	1.27 [1.10, 1.46]			
		Higher	No	1.21 [1.00, 1.47]	1.14 [0.90, 1.45]	13.8 [−11.5, 44.5]	0.287
			Yes	1.38 [1.20, 1.59]			
	Home	Lower	No	0.76 [0.61, 0.95]	1.14 [0.87, 1.49]	13.9 [−14.9, 49.0]	0.343
			Yes	0.87 [0.74, 1.02]			
		Higher	No	1.04 [0.85, 1.27]	0.91 [0.71, 1.17]	−10.1 [−41.8, 17.1]	0.458
			Yes	0.95 [0.81, 1.10]			
Italian	Away	Lower	No	0.91 [0.75, 1.11]	1.06 [0.84, 1.36]	6.4 [−19.8, 35.6]	0.616
(*n* = 1028)			Yes	0.97 [0.84, 1.12]			
		Higher	No	0.92 [0.76, 1.12]	1.21 [0.95, 1.53]	20.9 [−4.9, 53.4]	0.117
			Yes	1.12 [0.97, 1.28]			
	Home	Lower	No	0.96 [0.79, 1.16]	0.80 [0.63, 1.03]	−24.3 [−58.9, 2.9]	0.083
			Yes	0.77 [0.66, 0.90]			
		Higher	No	0.97 [0.80, 1.17]	0.91 [0.72, 1.16]	−9.3 [−39.3, 16.5]	0.469
			Yes	0.88 [0.76, 1.03]			
Total	Away	Lower	No	0.90 [0.79, 1.01]	0.99 [0.86, 1.15]	−0.6 [−16.6, 15.2]	0.939
(*n* = 3330)			Yes	0.89 [0.82, 0.97]			
		Higher	No	1.04 [0.93, 1.17]	1.02 [0.89, 1.18]	2.4 [−12.5, 18.1]	0.738
			Yes	1.07 [0.99, 1.16]			
	Home	Lower	No	0.84 [0.75, 0.96]	0.82 [0.70, 0.96]	−21.8 [−41.9, −4.5]	0.012
			Yes	0.69 [0.63, 0.76]			
		Higher	No	0.98 [0.87, 1.11]	0.85 [0.73, 0.98]	−18.2 [−36.7, −2.1]	0.025
			Yes	0.83 [0.76, 0.91]			

Abbreviation: 95% confidence intervals in brackets. *n* = number of observations; $\tilde{\mu }$ = expected mean number of cards received; $e^{\beta }$ = factor change in the expected mean number of cards received when moving from playing without spectators to playing with spectators, holding other variables constant; % = percentage change in the expected mean number of cards received when moving from playing without spectators to playing with spectators, holding other variables constant; *p* = *p*-value for *z*-test.
